# Ruthenium Oxide Nanorods as Potentiometric pH Sensor for Organs-On-Chip Purposes

**DOI:** 10.3390/s18092901

**Published:** 2018-09-01

**Authors:** Esther Tanumihardja, Wouter Olthuis, Albert van den Berg

**Affiliations:** BIOS Lab on a Chip Group, Technical Medical Centre, MESA + Institute for Nanotechnology, and Max Planck Center for Complex Fluid Dynamics, University of Twente, 7522 NB Enschede, The Netherlands; w.olthuis@utwente.nl (W.O.); a.vandenberg@utwente.nl (A.v.d.B.)

**Keywords:** ruthenium oxide, potentiometric sensor, pH sensor, organs-on-chip, lab-on-chip

## Abstract

A ruthenium oxide (RuOx) sensor for potentiometric pH sensing is currently being developed for organs-on-chip purposes. The sensor was fabricated from a Ru(OH)_3_ precursor, resulting in RuOx nanorods after heating. An open-circuit potential of the RuOx electrode showed a near-Nernstian response of −58.05 mV/pH, with good selectivity against potentially interfering ions (lithium, sulfate, chloride, and calcium ions). The preconditioned electrode (stored in liquid) had a long-term drift of −0.8 mV/h, and its response rate was less than 2 s. Sensitivity to oxygen was observed at an order of magnitude lower than other reported metal-oxide pH sensors. Together with miniaturizability, the RuOx pH sensor proves to be a suitable pH sensor for organs-on-chip studies.

## 1. Introduction

Organs-on-chips are a new, in vitro model of human tissue, where cells are cultured and perfused in microfluidic devices. The devices can be configured to mimic cells’ microenvironments as realistically as possible (in terms of physical/chemical cues, tissue–tissue interfaces, and perfusion). This way, the cells can replicate human physiology and pathology at organ-level, thus providing an improved model to the too-simplistic 2D (or even 3D) cell culture. Applications of organs-on-chip as an alternative to the animal models are thus more ethical, physiologically representative/predictive, and also personalizable [1,2,3].

Many different kinds of organs-on-chips have been developed over the years [3]. Typically, the cells have been cultured in micrometer-size chambers or channels, leaving low accessibility to probe or monitor the cells from outside the chip. The small dimensions also mean that very minute amounts of medium is involved, making offline sampling also limited. Consequently, microsensors can be of great value in providing readouts for organs-on-chip studies. Miniaturized sensors can be placed close to the cells, enabling accurate non-invasive online monitoring of minute analytes.

This work explores on-chip pH sensors, which are useful for long-term cell-culture/differentiation and tissue studies. Metal oxide is a well-known class of on-chip pH sensors for its robust, inert, and miniaturizable properties. Although the metal oxides (where the most commonly investigated ones are SbO_2_, TiO_2_, CuO, IrO_2_, and RuO_2_) show a Nernstian response to proton concentration [4], metal oxide electrodes often suffer from sensitivity to other oxidizing/reducing agents [4,5]. An especially critical and unavoidable oxidizing agent for organs-on-chip studies is dissolved O_2_, where it often acts as an operational parameter. Studies on IrO_2_, for instance, have reported a potential response as much as 80 mV going from a deoxygenated to oxygenated buffer [6,7]. Ruthenium oxide (RuOx), however, might be an exception to this shortcoming [4,8], and is therefore the focus of this work.

RuOx is proton-sensitive due to the reversible redox equilibrium between two different solid phases/oxidation states of the metal oxides, in which proton is involved [8,9,10]:

RuO_2_·2H_2_O + H^+^ + e^−^ ↔ H_2_O + Ru(OH)_3_ (1)

An adsorbed proton leads to the attraction of an electron through this conducting oxide, resulting in the reduction of the Ru ion. This leads to a net potential change, generating measurable change in an open-circuit potential.

Recent years have seen many works on the RuOx pH sensor for different applications. Table 1 summarizes the recent works on the RuOx pH sensor, as well as their results and applications. While pH sensitivity, range, drift, and response time have commonly been characterized, very few have touched on oxygen sensitivity. Lonsdale et al. [11] described the elimination of oxygen sensitivity through the addition of a Ta_2_O_5_ layer on RuO_2_. Xu et al. [12] showed there to be a high resemblance between calibration curves of RuO_2_-MWCNTs measuring in oxygen-saturated and nitrogen-saturated buffers. However, there no studies have been done on characterized oxygen sensitivity of RuO_2_ in organs-on-chip configuration so far.

In this work, the RuOx electrode was fabricated from a Ru(OH)_3_ precursor, which was precipitated from Ru^3+^ and OH^−^ ions. The precursor was heated to form RuO_2_ with nanorods morphology. The same electrode was intended to be developed into a dual sensor—a potentiometric pH sensor and amperometric nitric oxide sensor, where the nanorods morphology is desired for the latter. Therefore, the same RuOx nanorods were studied as a pH sensor, although the morphology did not theoretically improve the potentiometric signal.

This contribution evaluates the suitability of RuO_2_ nanorods as an on-chip pH sensor in novel organs-on-chip settings. Its drift behavior, selectivity, response time, and oxygen sensitivity are presented and discussed. The first organs-on-chip application for the RuOx pH sensor envisions a hypoxia study of cardiomyocytes. In a hypoxic condition, cardiomyocytes undergo anaerobic glycolysis which results in a drop in pH as much as 1–2 pH units. During the study, oxygen levels were expected to vary from fully oxygenated to deoxygenated. Applicability of the sensor is currently being assessed with this application in mind.

## 2. Materials and Methods

### 2.1. Electrode Fabrication

The RuOx electrode was fabricated according to the protocol of Chen et al. [16] RuCl_3_·*n*H_2_O (Aldrich, Saint Louis, MO, USA, 99.98%) was dissolved in de-ionized water to make 10 mL of 5 mM RuCl_3_ solution. 5 mM NaOH (Aldrich, 98%) solution was then added drop by drop to the solution, until the solid Ru(OH)_3_ precursor precipitated. Precipitation typically occurred around pH 4 (from an initial pH of around 2). The precursor was then isolated by centrifugation and suspended in DI water. The resuspension was spread onto clean substrate (sputtered circular Pt electrode, 2.4 mm in diameter, on glass chip), left to dry in room temperature, and then heated to 350 °C in a preheated oven (atmospheric environment) for 3 h. RuO_2_ nanorods were formed after the heat treatment, as confirmed by scanning electron microscopy (SEM) imaging.

Pt electrodes on glass chips were first electrochemically cleaned before being used as RuOx substrate by applying cyclic potential sweeps (20 times with a scan rate of 100 mV/s in 0.5 M H_2_SO_4_ between −0.6 and 1 V (vs. Ag/AgCl), ending in 1 V).

### 2.2. Experimental Setup

The modified chip with grown RuOx nanorods was used for most experiments, unless stated otherwise. The glass chip was used with a Teflon chip holder, made in-house. The chip holder exposed the electrodes’ active areas to the electrolyte chamber. All potentials were measured against a liquid-junction Ag/AgCl (satd. KCl) reference electrode (CH Instruments), typically placed ~5 mm from the RuOx electrode. The setup was placed inside a Faraday cage during all measurements. All measurements were carried out using a Bio-Logic SP300 bipotentiostat (input impedance of >100 GΩ) and were performed at room temperature of around 22 °C.

### 2.3. Measurement Protocol

Open-circuit potential measurements were performed in buffer solutions. pH 4 used a potassium hydrogen phthalate buffer, pH 5 acetic acid/sodium acetate buffer, pH 7 disodium hydrogen phosphate/potassium hydrogen phosphate buffer, pH 8 phosphate buffered saline, and pH 10 boric acid/sodium hydroxide buffer. The pH of the solutions was confirmed with a Mettler-Toledo SevenMulti pH meter, calibrated with standard buffer solutions of pH 4, 7, and 10.

For the pH response experiment, the open-circuit potential was recorded for 150 s at 1 s intervals. The last point was taken as a potential response to determine pH sensitivity. Interference experiments were done in air-saturated pH 7.4 phosphate-buffered saline (PBS) buffer. Ions of different concentrations were dissolved in the buffer and measured.

Longer measurements in air-saturated pH buffer were done for the drift experiment. Drift was calculated using the linear-fit slope of the potential over the measurement period.

Similar recording was also performed for the response-time experiment. The open-circuit potential was initially recorded in a 5 mL stirred solution of 20 mM KH_2_PO_4_, 137 mM NaCl, and 5 mM KCl. The solution was stirred with a magnetic stirrer hovering above the electrode. Over time, different amounts of 0.5 M K_2_HPO_4_ were added (by pipetting) to change the proton concentration by 0.6 pH units (except for the first addition, which resulted in a change of 1.2 pH units due to the unbuffered nature of the initial solution). Response time was deduced by linear fitting of the slope and calculating the intersecting time points between the stable potential plateau and the slope.

The oxygen sensitivity measurement was done in a pH 7 buffer, which had been purged with N_2_ gas for 1 h. Open-circuit measurement began with the buffer still purged with N_2_ gas. After ~4 h of measurement, the N_2_ gas was stopped and the buffer solution purged with instrument air (compressed, filtered air, free of contaminates) instead. The measurement was left to record for another ~4 h.

## 3. Results

### 3.1. Fabrication and Characterization

**Figure 1:**
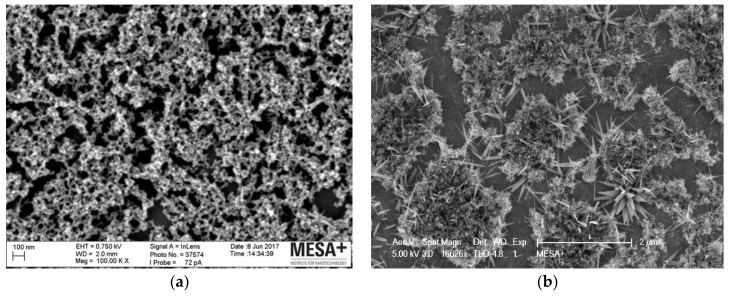
Scanning electron microscopy (SEM) images of the ruthenium oxide (RuOx) electrode: (**a**) Precipitated Ru(OH)_3_ precursor, isolated and spread onto a silicon surface; (**b**) heat-treated precursor (at 350 °C, for 3 h) on a platinum electrode surface formed RuO_2_ nanorods with a width of around 50 nm and length of 200–350 nm.

Every heat-annealed RuOx was imaged by SEM to confirm the creation of nanorods. An example of the produced nanorods sample is given in Figure 1b. As a comparison, Figure 1a shows the freshly precipitated precursor on silicon. As can be seen, the heat treatment did not convert all amorphous precursor into nanorods. The conversion seemed to depend on a few different parameters, most notably the substrate material and cleanness, as well as the presence of organics during the heat treatment. On (electrochemically) cleaned Pt substrate and in the absence of organics during heat treatment, nanorods typically grew (rather sparingly) on the precursor, as shown in Figure 1b. Most of the nanorods were around 50 nm wide and 200–350 nm long, forming a pointy rod shape as its width tapers along the length.

RuO_2_ stoichiometry was confirmed using energy-dispersive X-ray spectroscopy (EDX). EDX measurement of annealed RuOx with a visible nanorods morphology sample (shown in Supplementary Information (SI) Figure S1) revealed a Ru to O ratio of approximately 1:2.

### 3.2. pH Sensitivity and Selectivity

**Figure 2:**
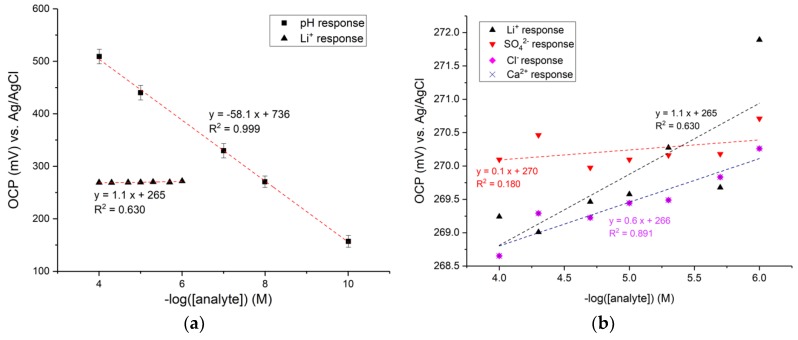
Experimental results of the RuOx electrode: (**a**) Open-circuit potential of the RuOx electrode in different pH buffer solutions. The error bar shows one standard deviation out of three different sets of measurement. Largest cross-sensitivity, towards lithium ions, is plotted as a comparison. Dashed lines are linear fitting through the points; (**b**) open-circuit potential of the RuOx electrode with the presence of different ions (lithium, sulfate, chloride, calcium ions) in pH 7.4 buffer. Dashed lines are linear fitting through the points.

The open-circuit potential of the RuOx electrode as a function of pH is shown in Figure 2. Figure 2a shows the typical potential response in air-saturated pH buffers from pH 4–10. The mean of the three different measurements (using three different electrodes) is plotted, with the error bar showing one standard deviation among the data. The slope of linear fitting through the measurement points indicates the electrode pH sensitivity of −58.1 ± 1.2 mV/pH, with extrapolated E^0^ of 736 ± 18 mV.

The RuOx electrode was also tested for its selectivity against possibly interfering ions, namely lithium, sulfate, chloride, and calcium ions. The potential response towards the different ions is given in Figure 2b. The highest sensitivity, taken from the slope of linear fitting through the points, was found towards lithium ions. This response of 1.06 mV/decade [Li^+^] is also plotted in Figure 2a as a comparison to the pH response.

**Figure 3:**
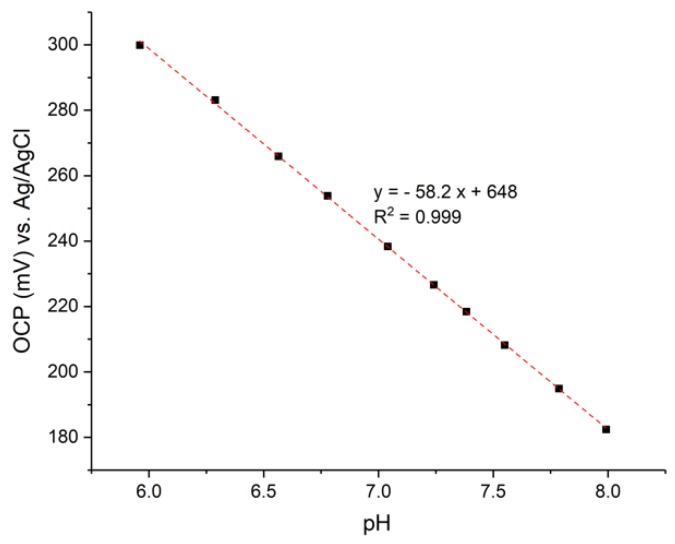
Open-circuit potential of (preconditioned) RuOx electrode in isotonic PBS buffer with a pH range of 6–8. Dashed line is linear fitting through the points.

The RuOx electrode was also tested for its pH response in a physiologically relevant milieu for future organs-on-chip applications. A solution comprising of 137 mM NaCl, 5 mM KCl, and 2 mM KH_2_PO_4_, which make up the inorganic constituent of human body fluids, was used. Different amounts of 0.5 M KHPO_4_ was added to the solution to vary the pH between 6 and 8, while making sure the solution was relatively isotonic to human body fluids. An open-circuit potential measurement of an RuOx electrode is plotted in Figure 3 as a function of the solution’s pH. A sensitivity of −58.23 mV/pH was estimated from linear fitting.

### 3.3. Drift and Aging

**Figure 4:**
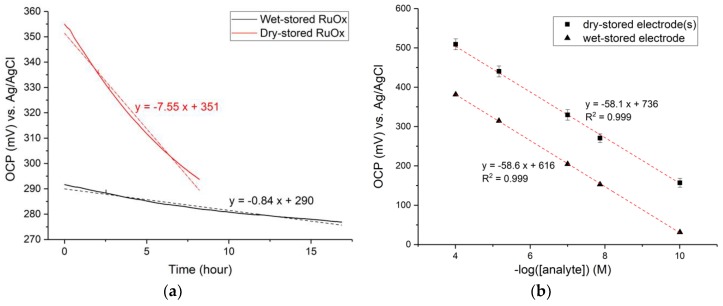
(**a**) Open-circuit potential of RuOx recorded over time in pH 7 buffer, fitted linearly (dotted lines). RuOx electrode showed high drift when stored in air (dry-stored) and significantly lower drift when stored in liquid (wet-stored); (**b**) comparison of open-circuit potential of wet-stored vs. dry-stored RuOx in different pH buffers, fitted linearly.

Drift behavior of the RuOx electrode was also studied. Freshly prepared or dry-stored electrodes were observed to undergo large short-term drift, decaying into a linear long-time drift after 8–10 h of measurement in a buffer. Preconditioning of the electrode, simply by storing it in liquid, allowed it to immediately attain the lesser long-term drift, as shown below in Figure 4a.

The open-circuit potential of RuOx electrode was measured over a long period of time in air-saturated pH 7 buffer, firstly after it had been stored in air (dry-stored RuOx graph). The measurement was then repeated with the same electrode after it had been stored in pH 7 buffer overnight (wet-stored RuOx graph). Dry-stored RuOx electrode showed a high drift of around −7.5 mV/h. Wet-stored RuOx electrode showed a significantly lower drift of −0.8 mV/h. The behavior was highly reversible, with the wet-stored electrodes again undergoing the large drift after drying (dry-blowing with N_2_). Furthermore, the wet-stored RuOx electrode performed comparably well to the freshly prepared/air-stored RuOx electrode in terms of pH sensitivity, as shown in Figure 4b.

At the time of writing, a number of RuOx electrodes had been used for as much as 10 months since their fabrication. The electrodes were stored in DI water for most of the time. So far, no detrimental effects on pH sensitivity, drift, or response time have been noticed from using, rinsing, and pH-cycling (between pH 2–10) the electrodes. Exposure of the electrodes to biological cell culture medium or bovine serum albumin also did not affect the electrode’s pH-sensing performance. pH response calibration after extensive exposure to culture medium and bovine serum albumin showed reproducible −58 mV/pH sensitivity.

### 3.4. Response Time

**Figure 5:**
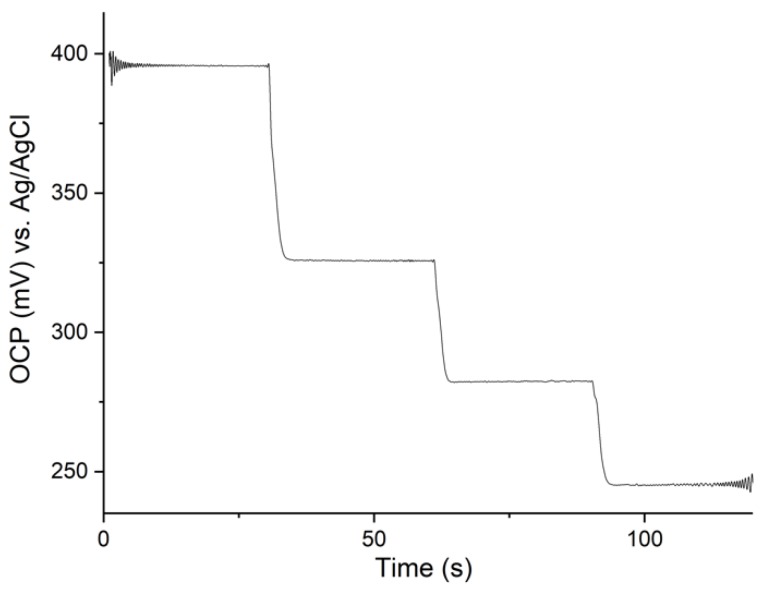
Open-circuit potential recording of the RuOx electrode in changing pH, low-pass filtered with cut-off frequency of ~40 Hz.

Filtered recording of RuOx open-circuit potential is shown in Figure 5, during which the solution pH was changed (the first change by 1.2 pH unit, the following by 0.6 pH unit). Due to the introduction of a magnetic stirrer in the Faraday cage during measurement, a noisy signal was acquired. For ease of analysis, a low-pass (cut off at ~40 Hz) and band-stop (cut off around the frequency of the magnetic stirrer, between 3 and 3.5 Hz) filters were applied, resulting in the graph shown in Figure 5. The raw signal can be seen in SI Figure S4. Analysis of the slopes resulted in a calculated response time between 2 and 3 s.

### 3.5. Oxygen Sensitivity

**Figure 6:**
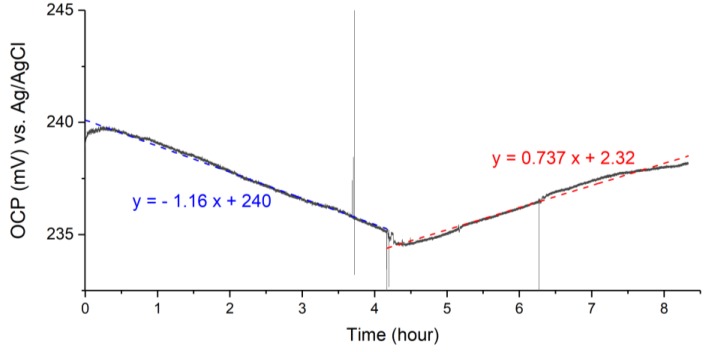
Open-circuit potential recording of wet-stored RuOx electrode, for the first 4 h in deaerated pH 7 buffer, followed by 4 h of reoxygenated pH 7 buffer. Fitted linearly by the dashed lines.

Open-circuit potential recording of the RuOx electrode in pH buffer with changing oxygen levels is shown in Figure 6. The negatively sloping potential recording in the first 4 h (linearly fitted with the blue dashed line) occurred in deaerated buffer solution and was of the same order of magnitude as the earlier observed wet-stored drift. A positive slope was observed in the following 4 h as reoxygenation took place in the buffer. Over the entire 4 h, the open-circuit potential drifted 3 mV as the buffer went from being deoxygenated to oxygenated. Large spikes in the recording were motion artefacts.

## 4. Discussion

### 4.1. pH Response and Selectivity

Near-Nernstian pH sensitivity was demonstrated by a number of different RuOx nanorods electrodes (Figure 2a). Based on the reaction given in Equation 1, the expected electrode potential at 25 °C in different pH can be expressed as [8,10]:

E = −0.059 V·pH + 0.740 V (vs. Ag/AgCl)(2)

The fitted experimental expression shown in Figure 2a (sensitivity of −58.05 mV/pH and extrapolated E^0^ of 0.73621 V) comes in very good agreement with the theoretical values, affirming the proposed pH-sensing theory.

A typical RuOx nanorods electrode surface (as shown in Figure 1b) exposes the annealed RuO_2_ nanorods, the amorphous Ru(OH)_3_ precursor, and the Pt surface. Presumably, the Pt surface would have an oxide layer, created both during the electrochemical cleaning process as well as during the heat treatment. PtO_2_ has also been reported as being a pH-sensitive metal oxide [4,17]. However, it is known that the potential pH response of PtOx is often lower than the Nernst-predicted −59 mV/pH [4,17]. It is also supported by our own observations of PtOx pH sensitivity of −34.46 mV/pH (SI, Figure S3). Therefore, it can be assumed that the observed near-Nernstian pH response is attributed to the RuOx modification.

As mentioned, the RuOx nanorods conversion seems to depend on several parameters. Unfortunately, the exact influence of the different parameters is not yet fully understood, which often challenges the reproducibility of the nanorods fabrication. While the typical resulting surface is shown in Figure 1b, different morphology of the nanorods has been observed across different fabrication batches. Nonetheless, the different morphology of RuOx nanorods does not seem to influence the potentiometric signal extensively. RuOx nanorods electrodes with different distributions and nanorods sizes performed comparably well in terms of their pH response.

The RuOx pH response was experimentally characterized between pH 4–10. Linear potential-pH response has been reported over a larger pH range for RuO_2_ [4,8,17]. However, the potential-pH graph (Pourbaix diagram) of ruthenium suggests that RuO_2_ becomes unstable in pH larger than 10 [9,18,19]. Keeping the organs-on-chip applications in mind, a closer investigation was done in the narrower, but more physiologically relevant, pH range instead.

Metal oxides are known to be readily complexed by different (an-)ions [8,18]. Operating in intricate (biological) milieu will inevitably expose the RuOx electrode to different, and possibly complexing or interfering, ions. Therefore, the effect of these ions on the electrode’s performance was studied. Relatively unchanging open-circuit potential in changing concentrations of different ions suggests the electrode was not extensively complexed by the ions in the solution, and is therefore sufficiently stable for the foreseen application. Residual sensitivity to the tested ions was so negligible that noise dominated the signal, resulting in poor signal-to-concentration linearity (shown by the low R^2^ values of linear fit).

The electrode response was also not interfered with when tested in pH buffers with (physiologically) isotonic osmolarity. The comparable near-Nernstian response in isotonic pH buffers suggests that good reproducibility can be expected of the RuOx nanorods electrodes when employed in biological milieu.

### 4.2. Drift and Aging

As mentioned, the RuOx electrode drifts differently when the electrode has been stored in air than when it has been stored in liquid. A large short-term drift was observed from freshly prepared or dry-stored RuOx electrodes, which decayed into a long-term drift of lower magnitude after a 8–10 h immersion in liquid. It was also demonstrated that electrodes stored/aged in liquid immediately showed a lower magnitude drift (Figure 4a). It could also be seen from the measurements (Figure 2a, Figure 3, and Figure 4) that this aging process resulted in a change of E^0^ value of around −60 to −100 mV. This drift decay and the shift in E^0^ value after the aging metal oxide electrode in liquid was reported across different metal oxide electrodes [6,7,8,10,20,21]. Literature has attributed this behavior either to the effect of hydration on the metal oxides’ surface [8,17], to redox processes involving atmospheric oxygen [7], or to the chemical modification of the hydroxide groups on the oxide surface [22]. Looking at the marginal response of the RuOx to changing oxygenation (Figure 6), it is unlikely that atmospheric oxygen alone caused the observed large drift. The work of McMurray et al. [8] also showed similar drift/aging behavior, albeit their electrode being described as completely indifferent to oxygen concentration. Given the highly reversible drift behavior, it is also unlikely that chemical modification caused the drift. The reversibility, however, is in line with the hydration phenomenon. Slow hydration could take place on the RuOx surface, introducing a change in surface energy, thus giving rise to the potential drift. It is also in line with the proposed sensing theory, where the equilibrium involves two hydrated forms of RuOx. Based on the observed drift behavior and in view of the literature, it can be reasonably concluded that the slow surface hydration is what gives rise to the observed drift.

The long-term drift of wet-stored RuOx electrode was observed to be −0.8 mV/h (Figure 4a), which corresponds to a ΔpH of 0.013 per hour. Such a potential drift can be sufficient for measurements over several hours in organs-on-chip applications. More accurate and longer measurements can also be achieved, should on-chip recalibration strategy be feasible to implement.

### 4.3. Response Time and Oxygen Sensitivity

The reaction time experiment suggested that the system reacted to the introduced pH change within 2 to 3 s. However, it was noticed that the resulting signal (particularly the slope) was highly dependent on how the solution was pipetted into the electrochemical cell. Keeping the setup and the protocol in mind, it can be assumed that the rate at which the pH change was practically introduced was the limiting rate. Therefore, it can be concluded that the sensor’s true response time is less than 2 s. Such a response time is more than sufficient for the foreseen organs-on-chip applications, where pH change does not tend to occur so rapidly.

The oxygen sensitivity experiment showed a potential drift as low as 3 mV going from deoxygenated to oxygenated buffer. The potential shift corresponds to a ΔpH of 0.05. This proved to be significantly lower than other reported metal oxide sensors. IrO_2_ has also been reported to drift as much as 80 mV, going from deoxygenated to oxygenated buffer [6,7]. The PdO electrode, which showed a pH sensitivity of −54 mV/pH in oxygenated buffer, is rendered non-functional in deoxygenated buffer [23]. Given the necessity to operate in a wide range of oxygen levels, the RuOx proves to be a strong contender for the pH sensor in organs-on-chip applications. The apparent pH drift of a 0.050 pH unit going from deoxygenation to oxygenation is sufficiently low to allow for the accurate online monitoring of pH even in an oxygen-fluctuating environment, e.g., during a hypoxia study.

## 5. Conclusions and Outlook

The RuOx nanorods electrode was successfully fabricated through heat annealing of the Ru(OH)_3_ precursor. The electrode showed a near-Nernstian response to a pH of −58 mV/pH, which can be elucidated by a reversible redox reaction involving protons. The electrode proved to be a suitable pH sensor for future organs-on-chip applications, namely in terms of selectivity, drift (−0.8 mV/h or 0.013 pH unit/hour), response time (less than 2 s), and oxygen sensitivity (ΔpH of 0.05 going from oxygenated to deoxygenated). Future work includes application of the RuOx nanorods pH sensor in a hypoxic cardiomyocytes study, as well as investigating the same RuOx nanorods sensor for its amperometric nitric oxide sensing abilities. For a hypoxia study application, current chips can readily be used together with a Transwell insert in the chip holder. Use of a solid-state quasi Ag/AgCl reference electrode and miniaturization of the RuOx electrode are planned for future integration within a micro-chamber/channel.

## Figures and Tables

**Table 1:** Summary of recent works on RuO_2_ pH sensors, their results, and applications.

Material	pH Range	pH Sensitivity (mV/pH)	Response Time (s)	Drift (mV/Hour)	Application	Ref
RuO_2_-Ta_2_O_5_	-	−55.3	5–136	7.2	pH sensing of common beverages	[11]
RuO_2_	1–10	−77.74	<20	93.3	Detection of Helicobacter pylori	[13]
RuO_2_	2–11	−56	60–120	-	-	[14]
Pt-doped RuO_2_	2–13	−58	1–2	0.002	Water quality monitoring	[15]
RuO_2_-MWCNTs	2–12	−55	<40	-	-	[12]
RuO_2_ nanorods	2–10	−58	<2	−0.8	Organs-on-chip	This work

-: not (quantitatively) characterized/specified.

## Supplementary Materials

The following are available online at http://www.mdpi.com/1424-8220/18/9/2901/s1, Figure S1: EDX result of annealed RuOx, emitted photo electrons characteristic to oxygen and ruthenium were detected; Figure S2: Open-circuit potential signal of RuOx in different pH buffers is plotted over time. Last OCP point is taken as potential response of the solution’s pH for plotting; Figure S3: Open-circuit potential of bare Pt electrode as a function of pH, dashed line is linear fitting through the points; Figure S4: The noisy raw signal (blue) from the response time experiment overlaid with the filtered signal (low pass, cut-off frequency of ~40 Hz and band-stop, between 3–3.5 Hz) (green). Inset shows a closer view of the slope area.
