# Supplementary material for: Ruthenium Oxide Nanorods as Potentiometric pH Sensor for Organs-On-Chip Purposes
Source: Sensors (Basel). 2018 Sep 1;18(9):2901. doi: 10.3390/s18092901 (PMC6163346; doi:10.3390/s18092901)
Supplement: Supplementary file 1 [file sensors-18-02901-s001.pdf]

Supplementary Information

Ruthenium oxide nanorods as potentiometric pH sensor for organs-on-chip purposes

Esther Tanumihardja [1,*], Wouter Olthuis [1] and Albert van den Berg [1]

[1] BIOS Lab on a Chip group, MIRA Institute for Biomedical Technology and Technical Medicine, MESA+ Institute for Nanotechnology, and Max Planck Center for Complex Fluid Dynamics, University of Twente; e.tanumihardja@utwente.nl

* Correspondence: e.tanumihardja@utwente.nl

S1. Energy-dispersive X-ray spectroscopy of annealed RuOx

Sample used was a clean silicon wafer on which $Ru(OH)_3$ precursor was spread, and heat-treated at 320°C for 3 hours. EDX was performed on area where nanorods were visible (visualization was aided by SEM). Ratio of measured Ru to O was calculated to be 1:1.75, which is close to the expected 1:2 Ru:O stoichiometry.

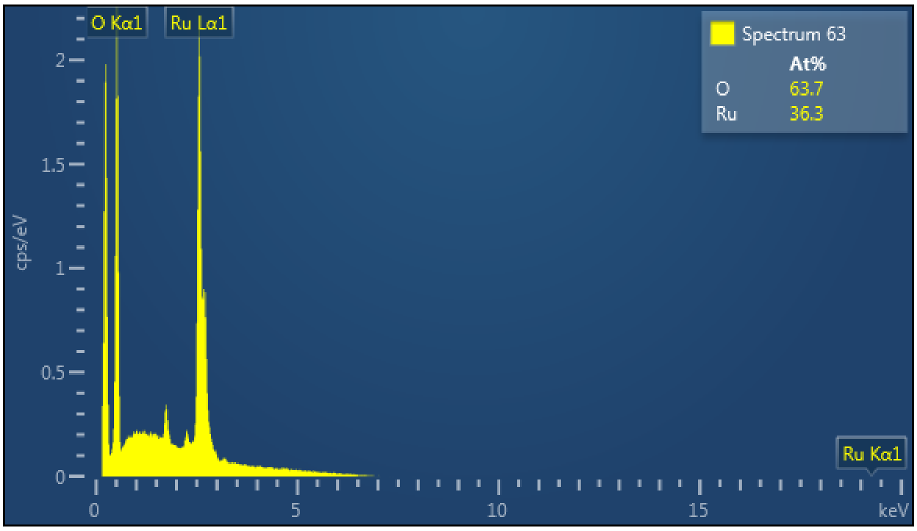

Figure S1. EDX result of annealed RuOx, emitted photo electrons characteristic to oxygen and ruthenium were detected.

S2. Time response RuOx

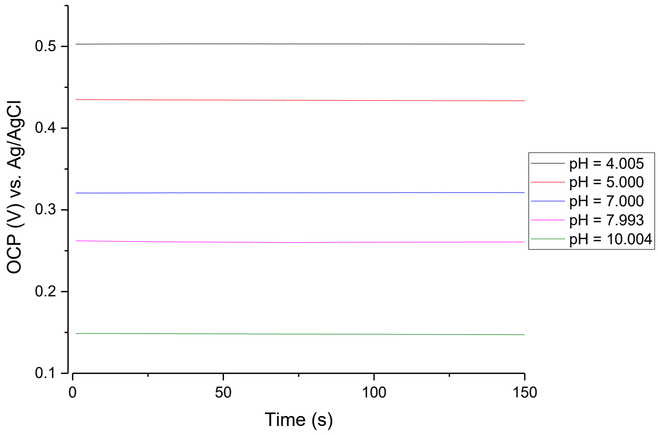

Figure S2. Open-circuit potential signal of RuOx in different pH buffers is plotted over time. Last OCP point is taken as potential response of the solution's pH for plotting.

S3. pH response of bare Pt electrode

Bare/unmodified Pt electrode on glass chip, also cleaned and heat-treated at 350°C for 3 hours, was tested for its pH response. Same pH buffers and protocol were used as RuOx pH response experiments.

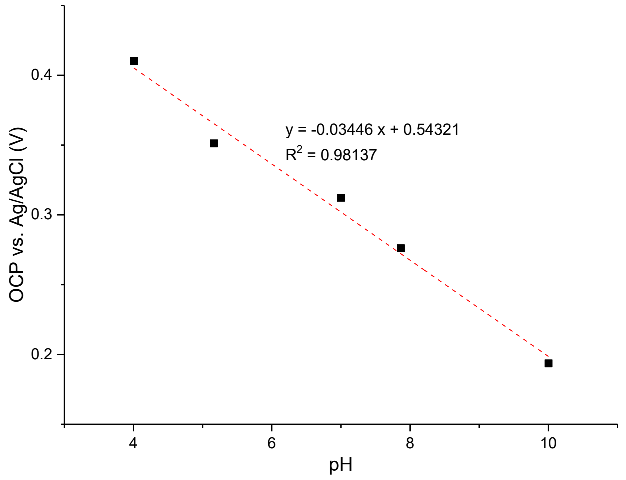

Figure S3. Open-circuit potential of bare Pt electrode as a function of pH, dashed line is linear fitting through the points.

S4. Response time raw result

Comparison of the raw and filtered response time experiment is given below. The overlaid graphs show that the filter did not alter information in the signal. Especially in the slope (inset), where filtering did not tamper with the response time analysis or the results thereof.

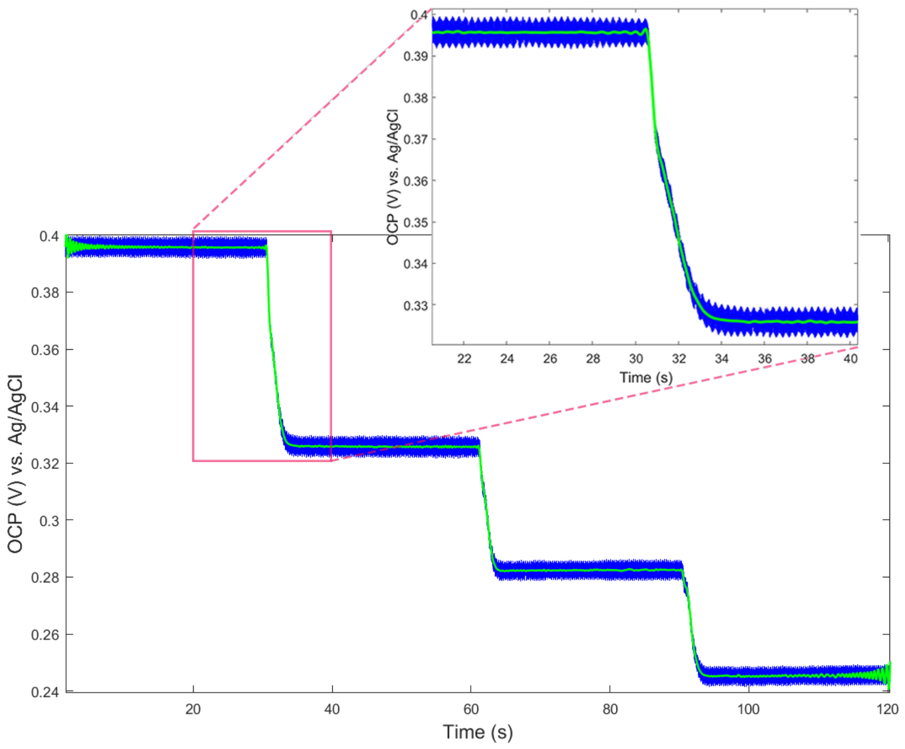

Figure S4. The noisy raw signal (blue) from the response time experiment overlaid with the filtered signal (low pass, cut-off frequency of ~40 Hz and band-stop, between 3-3.5 Hz) (green). Inset shows a closer view of the slope area.
